# Prenatal Identification and Molecular Characterization of Two Simultaneous De Novo Interstitial Duplications of Chromosomal Regions 7p22.1p21.1 and 15q24.1

**DOI:** 10.1155/2018/1513534

**Published:** 2018-02-11

**Authors:** Sabrina C. Burn, Kali Swift, Maria Palmquist

**Affiliations:** ^1^Department of Obstetrics, Gynecology & Women's Health, University of Minnesota, Minneapolis, MN, USA; ^2^Sanford School of Medicine, The University of South Dakota, Sioux Falls, SD, USA; ^3^Division of Maternal Fetal Medicine, Avera McKennan Hospital & University Health Center, Sioux Falls, SD, USA

## Abstract

The occurrence of simultaneous de novo chromosomal aberrations is extremely rare. Here, we describe two, previously unreported, simultaneous de novo interstitial duplications of chromosomes 7p and 15q. Amniocentesis was completed for a healthy gravida 4 para 3 woman due to her advanced maternal age and concurrent ultrasound findings of partial vermian agenesis, choroid-plexus cysts, and hypoplastic nasal bone. Cytogenetic analysis of cultured amniocytes by conventional chromosome analysis, comparative genomic hybridization, and fluorescence in situ hybridization revealed two interstitial duplications of the chromosomal regions 7p22.1p21.1 and 15q24.1, leading to partial trisomy of 7p and 15q and karyotype 46,XY,dup(7)(p22.1-p21.1),dup (15)(q24.1). Parental chromosomal analysis did not identify any heritable changes, suggesting both mutations were de novo in nature. Postnatal examination of the neonate was significant for low set ears, thick helices, flat nasal bridge, ankyloglossia, and aberrant head shape and size concerning for craniosynostosis. Postnatal MRI was consistent with Dandy-Walker variant showing hypogenesis of the inferior cerebellar vermis. To our knowledge, there are no prenatal or postnatal reports of comparable duplications involving these two regions simultaneously. Continued observation of the neonate may reveal further phenotypic consequences of these two simultaneous de novo interstitial duplications.

## 1. Introduction

It is well documented that the probability of chromosomal aberrations increases significantly with maternal age. An abnormal ultrasound accompanying advanced maternal age is significant and is most often followed by further genetic testing. The implementation and complementary use of conventional and newer molecular cytogenetic techniques have enabled diagnostic and prognostic parental counseling. As these techniques have steadily improved it is now possible to locate and characterize the aberration and identify potential consequences of such mutations. However, it is still difficult to provide adequate counseling in the absence of any prenatal or postnatal reports of similar chromosomal aberrations or any previously identified phenotypes. This is true in particular for previously unidentified and uncharacterized de novo interstitial duplications whose occurrence is relatively rare. Here, we report the prenatal identification and molecular characterization of two, previously unreported, simultaneous de novo interstitial duplications of chromosomal regions 7p22.1p21.1 and 15q24.1 with associated phenotype using a combination of advanced ultrasonography, conventional chromosome analysis, microarray comparative genomic hybridization (CGH), and fluorescence in situ hybridization (FISH) analysis.

## 2. Clinical Report and Cytogenetic Analysis

### 2.1. Initial Examination

**Figure 1:**
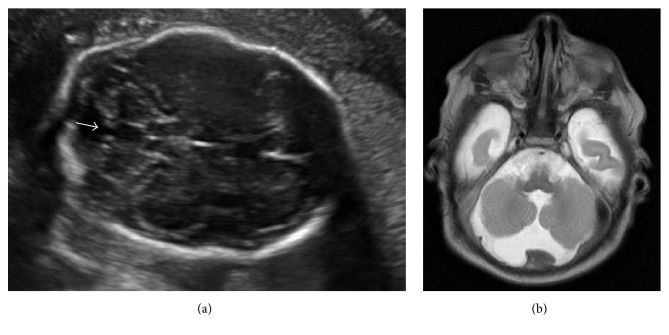
(a) Prenatal axial ultrasound shows the skull shape aberrancy and the presence of vermian agenesis with communication between the 4th ventricle and the cisterna magna (depicted by arrow). (b) Postnatal MRI was significant for hypogenesis of the inferior cerebellar vermis with mild fourth ventricular enlargement which communicates with a retrocerebellar CSF collection.

An otherwise healthy 37-year-old, gravida 4 para 3, woman was referred to our center after the identification of choroid-plexus cysts and concern for additional brain anomaly arose on her 20-week ultrasound scan. Complete sonographic examination at 21-week gestation identified multiple abnormalities, including partial vermian agenesis (formerly referred to as Dandy-Walker variant), hypoplastic nasal bone, choroid-plexus cysts, and dangling choroid (Figure 1(a)). The identification of multiple fetal anomalies suggested an increased likelihood of aneuploidy or other genetic abnormalities. The patient's advanced maternal age and brother with Down syndrome further indicated the possibility of a cytogenetic abnormality. Due to these factors, the patient elected to proceed with genetic amniocentesis.

### 2.2. Amniocentesis and Conventional Chromosome Analysis

Amniocentesis results were as follows: (i) Precision Panel analysis was performed via the Luminex xMap Technology of 20 known genetic syndromes and regions to identify the presence of aneuploidies in chromosomes 13, 18, 21, X, and Y. The Panel identified no abnormalities in the DNA loci tested. (ii) Conventional chromosomal analysis on amniotic fluid revealed an abnormal 46,XY,add(7)(p15) karyotype. More specifically, an abnormal chromosome complement characterized by the presence of additional quantifiable material of unknown origin was identified at band 7p15 in each metaphase cell analyzed.

### 2.3. Microarray Comparative Genomic Hybridization (CGH) Analysis

To further characterize the breakpoint region, estimate size, and approximate number of genes located within the 7p15 region, a microarray CGH analysis was performed. Analysis of chromosome 7 confirmed the presence of an interstitial gain of the 7p22.1p21.1 region with genomic coordinate chr7:5262454-20572298. The gain was approximately 15.31 Mb in size. Genoglyphix® Analysis software suggests this area of duplication is comprised of at least 72 genes, of which 43 are OMIM genes. The result is a clinically relevant alteration, which results in partial trisomy of 7p22.1p21.1. Unexpected was the detection of another clinically significant interstitial gain in chromosome 15. The interstitial gain was found in the 15q24.1 region with genomic coordinate chr15:73775826-75162902 and was approximately 1.39 Mb in size. This region contains approximately 31 genes, including 22 OMIM genes. Based on the size and content, this copy gain is expected to be clinically relevant and results in an additional trisomy of the 15q24.1 region.

### 2.4. Fluorescence In Situ Hybridization (FISH) Analysis

Results of Microarray CGH analysis were confirmed by FISH. FISH analysis of metaphase cells using BAC probes from 7p21.1 (RP11-5G13) and 7p2.1 (RP11-425P5) confirmed the presence of a duplication in the 7p22.1p21.1 region. Metaphase FISH excluded unbalanced translocation. Additional FISH analysis of interphase nuclei using a BAC probe from 15q24.1 (RP11-414J4) confirmed a duplication of the 15q24.1 region.

### 2.5. Parental Chromosomal Analysis

Subsequent chromosomal analysis of both parents was also completed to determine whether the two interstitial chromosomal gains were de novo in the fetus or from heritable duplication or translocation from a parent. Metaphase FISH analysis revealed normal karyotypes for both parents, with no genetic duplication in either the 7p22.1p21.1 or the 15q24.1 region, suggesting that the duplications identified in the fetus were de novo in origin.

### 2.6. Pregnancy Progression and Postnatal Development

After parental counseling on cytogenetic testing results, the patient elected to continue with the pregnancy. Follow-up imaging showed appropriate fetal growth and additional findings of abnormal calvarial shape suggestive of craniosynostosis and frontal bossing. Fetal echocardiogram at 25-week gestation showed no obvious structural abnormalities.

The pregnancy progressed to 39.2-week gestation, at which time the patient was scheduled for an induction of labor. Patient delivered a male neonate weighing 3,120 g (6 Ibs 14 oz). Infant had APGAR scores of 7 and 9 at one and five minutes of life, respectively. The neonate was transitioned into the Neonatal Intensive Care Unit for evaluation. Initial examination of the neonate revealed low set ears with thick helices, flattened nasal bridge, and ankyloglossia. Although the infant's head shape and size were concerning for craniosynostosis, no formal diagnosis was made. MRI, performed on day two of life, was consistent with partial vermian agenesis and showed hypogenesis of inferior cerebellar vermis with mild fourth ventricular enlargement which communicated with a retrocerebellar cerebrospinal fluid collection (Figure 1(b)). Patient and neonate were discharged on day three of life. At 4 months of age, it became evident that the infant had sensorineural hearing loss of the left ear with unrestricted hearing of contralateral ear, hypotonia, strabismus, and developmental delay. Denver Testing at 9 months of age further characterized the delay. Infant was unable to roll over, had not begun to work for a toy, and did not attempt to feed self. Continued evaluation of the infant throughout development will provide further clarification of the clinical significance and prognosis of these two simultaneous duplications.

## 3. Discussion

Prenatal microarray CGH and FISH analysis of the fetus allowed for the detection of two, previously unreported, simultaneous de novo interstitial duplications resulting in karyotype 46,XY,dup(7)(p22.1-p21.1),dup (15)(q24.1) and partial trisomy of 7p22.1p21.1 and 15q24.1. FISH analysis of the parents demonstrated that both abnormalities were de novo rather than heritable changes.

To our knowledge, the partial trisomy of 7p22.1p21.1 region seen in the neonate has not been described before. The first report on 7p interstitial duplication characterized by array CGH was published by Chui and colleagues [1]. Reported findings regarding other 7p duplications, without involvement of additional chromosomes, describe variable phenotypes, with common features including intellectual disability, hypotonia, craniofacial dysmorphism, skeletal abnormalities, and cardiovascular malformations [1–4]. More specifically, findings suggest that 7p21 is a critical region for craniofacial dysmorphism and skeletal development [3]. There has been one reported syndrome, Saethre-Chotzen, which is one of the most common autosomal dominant disorders associated with craniosynostosis and is also associated with other craniofacial and limb anomalies. Saethre-Chotzen syndrome maps to locus 7p21p22 and is caused by a mutation in the TWIST gene, which encodes a basic helix-loop-helix transcription factor [5, 6]. TWIST mutations have been described as a result of both duplications and deletions within the gene. These mutations result in impairment of head mesenchyme induction by TWIST and therefore result in craniosynostosis [6]. While there is limited data showing that an increase in the copy number of the TWIST gene may result in craniosynostosis, it is intriguing to speculate that the observed duplications of the 7p22.1p21.1 region and the corresponding doubling of the copy number of the TWIST gene may lead to similar aberrant gene function and cranial phenotype as reported for the TWIST mutations. When combined, these reports document the potential consequence such a large duplication in 7p may have on the developing infant.

The simultaneous duplication of 15q24.1 overlaps a well described microdeletion/microduplication region, 15q24.1q24.2, of chromosome 15. However, what is unique about this case is that the gain identified does not include the entire characterized region commonly described as the smallest region of overlap (SRO). In addition, the duplication breakpoints do not localize to previously described locus control regions (LCRs). For comparison purposes, the proximal duplication breakpoint found in this patient is between 15q24A (also referred to as BP4) and 15q24B (BP1), with the distal breakpoint between 15q24B (BP1) and 15q24C as shown by El-Hattab and collaborators [7, 8]. To date, there have been 15 reported patients with microdeletions and 2 patients with microduplications within this region. The patients with microduplication exhibited a phenotype significant for cognitive impairment, skeletal deformities, and dysmorphic facial features [9]. Together, these reports indicate the potential phenotypical outcomes of such a mutation.

Postnatal evaluation of the neonate has shed some light onto the possible phenotype that may result from these two simultaneous de novo duplications of chromosomal regions 7p22.1p21.1 and 15q24.1. The neonate displayed various craniofacial anomalies including low set ears with thickened helices, flattened nasal bridge, ankyloglossia, and abnormal calvarial shape. Previously published case studies reporting on single duplications in these two chromosomal regions described similar craniofacial anomalies. MRI did confirm the presence of partial vermian agenesis, which can be caused by various genetic anomalies. As the neonate has continued to develop there is additional evidence of developmental delay and unilateral sensorineural hearing loss.

In conclusion, the combination of conventional cytogenetic and molecular cytogenetic analysis and sonographic imaging provided valuable prenatal data on a developing fetus. Microarray CGH and FISH analysis allowed for the identification and characterization of two, previously unreported, simultaneous de novo interstitial duplications of chromosomal regions 7p22.1p21.1 and 15q24.1, resulting in the partial trisomy of 7p22.1p21.1 and 15q24.1. This case well illustrates that microarray CGH and FISH analyses are powerful cytogenetic tools in prenatal diagnosis. It was challenging, however, to provide comprehensive and definitive counseling to the parents on long-term prognosis and perspectives of their infant in the absence of any previous reports documenting genetic aberrations precisely in these two chromosomal regions. Continued observation and monitoring of the infant may provide further insights into the phenotype and the effects caused by these two simultaneous de novo interstitial duplications.
